# Synonymous Codon Usage in TTSuV2: Analysis and Comparison with TTSuV1

**DOI:** 10.1371/journal.pone.0081469

**Published:** 2013-11-26

**Authors:** Zhicheng Zhang, Wei Dai, Dingzhen Dai

**Affiliations:** 1 Department of Animal Science and Technology, Jinling Institute of Technology, Nanjing, China; 2 Key Laboratory of Zoonoses of Anhui Province, Anhui Agricultural University, Hefei, China; Centers for Disease Control and Prevention, United States of America

## Abstract

Two species of the DNA virus Torque teno sus virus (TTSuV), TTSuV1 and TTSuV2, have become widely distributed in pig-farming countries in recent years. In this study, we performed a comprehensive analysis of synonymous codon usage bias in 41 available TTSuV2 coding sequences (CDS), and compared the codon usage patterns of TTSuV2 and TTSuV1. TTSuV codon usage patterns were found to be phylogenetically conserved. Values for the effective number of codons (ENC) indicated that the overall extent of codon usage bias in both TTSuV2 and TTSuV1 was not significant, the most frequently occurring codons had an A or C at the third codon position. Correspondence analysis (COA) was performed and TTSuV2 and TTSuV1 sequences were located in different quadrants of the first two major axes. A plot of the ENC revealed that compositional constraint was the major factor determining the codon usage bias for TTSuV2. In addition, hierarchical cluster analysis of 41 TTSuV2 isolates based on relative synonymous codon usage (RSCU) values suggested that there was no association between geographic distribution and codon bias of TTSuV2 sequences. Finally, the comparison of RSCU for TTSuV2, TTSuV1 and the corresponding host sequence indicated that the codon usage pattern of TTSuV2 was similar to that of TTSuV1. However the similarity was low for each virus and its host. These conclusions provide important insight into the synonymous codon usage pattern of TTSuV2, as well as better understangding of the molecular evolution of TTSuV2 genomes.

## Introduction

It is well known that the 64 codons of the genetic code encode the 20 standard amino acids as well as three translation termination signals (UAA, UAG, UGA). Each amino acid is encoded with at least one codon (e.g., Met and Try); however, due to the degeneracy of the genetic code, some amino acids are encoded with up to six codons (e.g, Leu, Ser and Arg). Codons encoding the same amino acid are referred to as synonymous codons. Studies have indicated that synonymous codon usage is non-random and species-specific [1]. Some synonymous codons are more frequent than others both within and between genes, and this phenomenon is termed synonymous codon usage bias [2]. In general, genome dynamics, primarily mutation pressure, facilitate the evolution of novel viruses and strains and contribute to adaption to environment and host [3]. Hence, codon usage variation is considered to be an indicator of the type of force that influences genome evolution. Investigation of codon bias and the forces that influence it provides insights into the fundamental mechanisms of viral evolution. Thus, understanding codon bias is essential to understand the interplay between a virus and its host. 

It was well established that mutational pressure and natural selection [4,5] were presented as the two major factors accounting for codon usage variation in mammalian, protozoan and endosymbiotic bacterial genes [6]. In their investigate of codon usage variation, Shackelton et al (2006) found that codon usage bias was strongly correlated with overall genomic GC content, indicating that compositional constraint under mutation pressure rather than natural selection was the main factor for specific codons [7]. Naya et al (2001) examined the *Chlamydomonas reinhardtii* genome, which has a high GC content, and found no evidence that base constraint under mutation pressure was responsible for determining the codon usage pattern [8]. Recently, it was also reported that codon usage variation is related to gene function and length [9,10], DNA replication and selective transcription [11], protein secondary structure [12,13] and environmental factors [14].

Torque teno virus (TTV) is a small, single-stranded, negative-sense non-enveloped, circular DNA virus [15], which has been classified as a member of the recently discovered *Anelloviridae* family [16]. It was first identified in a Japanese patient with post-transfusion hepatitis of unknown aetiology in 1997 [17]. Subsequently, TTV has been detected in humans, chimpanzees, poultry, swine, cattle, sheep, cats and dogs [18,19]. TTV was first detected in swine in 1999 and two genetically distinct species, Torque teno sus virus 1 (TTSuV1) and 2 (TTSuV2), have been identified based on the low sequence identity between the two variants [20]. 

Recently, Torque teno sus virus (TTSuV) infection of pigs has become widespread in many countries, including the USA, Canada, Spain, Germany, China, Japan, Korea and Brazil [21]. Despite the fact that TTV infection in humans is not yet directly associated with any disease [22], TTSuVs have been shown to be involved in co-infection with other diseases, including the experimental induction of porcine dermatitis and nephropathy syndrome in combination with porcine reproductive and respiratory syndrome virus infection [23] and post-weaning multisystemic wasting syndrome (PMWS) in combination with porcine circovirus type 2 (PCV2) infection in a gnotobiotic pig model [24]. Moreover, Kekarainen et al. (2006) found that TTSuV2 was detected at a significantly higher rate in PMWS pigs than in healthy pigs [25]. Other research comfirmed that the replication of TTSuV2, but not of TTSuV1, was up-regulated in the pigs with PMWS [26,27]. This result was supported by Taira et al (2009), who examined animals suspected of infection with PMWS and porcine respiratory disease complex [28]. However, due to the limited number of animal species examined and the lack of information about viral cell and tissue tropism, the characteristics and evolution of TTSuV are not fully understood. 

We previously investigated synonymous codon usage in TTSuV1 [29] and began to suspect that this method might be important for elucidating the molecular mechanism and evolutionary process of TTSuV. In this study, synonymous codon usage bias was analyzed in the coding sequences (CDS) from the 41 available TTSuV2 genomes, and the codon usage patterns of TTSuV2 and TTSuV1 were compared.

## Materials and Methods

### Sequences data

Complete genome sequences from 41 TTSuV2 isolates were downloaded from the National Center for Biotechnology Information (http://www.ncbi.nlm.nih.gov/Genbank/). Each TTSuV2 CDS was analyzed using DNAStar version 7.1 (DNAStar, Madison, WI). Table 1 summarizes relevant details about these viral sequences.

**Table 1:** 41 complete TTSuV2 genes used in this study.

**No.**	**Accession no.**	**Name**	**Isolation**	**Year**	**Length(bp)**
1	AY823991	2p	Brazil	2005	1875
2	GU188046	472142	Germany	2008	1878
3	GU456385	PTTV2b-VA	USA	2008	1878
4	GU456386	PTTV2c-VA	USA	2008	1878
5	GU570197	TTV2_GE9	Spain	2011	1884
6	GU570203	TTV2_1907	Spain	2011	1884
7	GU570204	TTV2_G31	Spain	2011	1884
8	GU570205	TTV2_G33	Spain	2011	1884
9	GU570206	TTV2_G43	Spain	2011	1875
10	GU570207	TTV2_G61	Spain	2011	1875
11	GU570208	TTV2_G64	Spain	2011	1884
12	GU570209	TTV2_GE1	Spain	2010	1884
13	HM633214	TTV2Bj7-2	China	2009	1863
14	HM633215	TTV2Bj2-3	China	2009	1884
15	HM633216	TTV2Bj4-3	China	2009	1884
16	HM633217	TTV2Bj6-2	China	2009	1875
17	HM633218	TTV2Bj6-3	China	2009	1875
18	HM633219	TTV2Bj7-3	China	2009	1884
19	HM633220	TTV2Fj2	China	2009	1884
20	HM633221	TTV2Jl1	China	2009	1884
21	HM633222	TTV2Jl2	China	2009	1884
22	HM633223	TTV2Jl27	China	2009	1863
23	HM633224	TTV2Bj1-2	China	2009	1878
24	HM633225	TTV2Hb1	China	2009	1884
25	HM633226	TTV2Bj8	China	2009	1863
26	HM633227	TTV2Bj11	China	2009	1878
27	HM633228	TTV2Bj12	China	2009	1875
28	HM633229	TTV2Gx1	China	2010	1872
29	HM633230	TTV2Gx2	China	2009	1878
30	HM633231	TTV2Gx3-2	China	2009	1884
31	HM633232	TTV2Gx4	China	2009	1872
32	HM633233	TTV2Jx1	China	2009	1884
33	HM633234	TTV2Jx2	China	2009	1875
34	HM633235	TTV2Ln13	China	2009	1863
35	HM633236	TTV2Ln14	China	2009	1872
36	HM633237	TTV2Ln21	China	2009	1863
37	HM633238	TTV2Ln22	China	2009	1863
38	HM633239	TTV2Ln23-2	China	2009	1875
39	HM633240	lung1	China	2009	1884
40	HM633241	lung3	China	2009	1878
41	HQ204188	SC	China	2010	1878

### Recombination analysis

The Recombination Analysis Tool (RAT, http://cbr.jic.ac.uk/dicks/software/RAT/) was used to detect recombination events in TTSuV2 and TTSuV1 sequences. Recombination is a prevailing drive that shapes genome evolution, and it is believed to influence the efficacy of natural selection on codon usage [30]. RAT uses a distance-method-based algorithm to perform pair-wise comparisons with multiple sequence alignments (DNA or protein). The RAT graph represents the genetic distance of each sequence in the alignment to a reference sequence (Y-axis) for each position in the sequence (X-axis). A putative recombination event is detected when the lines representing two sequences intersect in the graph [31].

### Compositional properties measures

General nucleotide composition (A%, C%, T% and G%) and nucleotide composition at the third position of each codon (A_3S_%, C_3S_%, T_3S_% and G_3S_%) were analyzed for TTSuV2 CDSs using Molecular Evolutionary Genetics Analysis (MEGA) software version 5.0 [32]. The GC and GC_3S_ index was used to calculate the overall G + C content in the gene sequence and at the third position of synonymous codon (excluding Met, Trp and termination codons). 

### Measure of synonymous codon usage

Relative synonymous codon usage (RSCU) values and effective number of codons (ENC) values were calculated using CodonW software version 1.4 (http://codonw.sourceforge.net). The RSCU is defined as the ratio between the usage frequency of one codon in the gene and its expected frequency in the synonymous codon family (i.e., the observed frequency of a codon adjusted for amino acid composition). RSCU value is calculated according to the following published equation [33]:

$$RSCUij=\frac{Xij}{\frac{1}{ni}\sum_{j=1}^{ni}Xij}$$

*X*_*ij*_ denotes the position of the codon (i) in the CDS for the corresponding amino acid (*j*). *n*_*i*_ denotes the total number of synonymous codons encoding the amino acid at this position. Codons with RSCU values greater than 1.0 exhibit positive codon usage bias, while those with RSCU values less than 1.0 have negative codon usage bias. RSCU values of 1.0 indicate that the codon frequencies are equal or random. 

The ENC is the most useful estimator of absolute synonymous codon usage bias [34] and can indicate the degree of synonymous codon bias in a codon family. ENC values range from 20 (only one synonymous codon occurs in the CDS) to 61 (all synonymous codons occur with equal frequency). A gene with an ENC value lower than 35 is generally considered to have significant codon usage bias. 

### Correspondence analysis

Correspondence analysis (COA), also known as principal component analysis, was performed with CodonW software version 1.4. COA is the most commonly used multivariate statistical analysis method [35]. In this analysis, COA was used to study the major trends in sequence variation and distribute genes along continuous axes according to these trends. Each gene was represented as a 59-dimensional vector, each dimension corresponding to the RSCU value for each sense codon (excluding Met, Trp and termination codons). Major variation trends within this dataset can be determined with the relative inertia: genes were positioned according to the major inertia to determine the major factors affecting codon usage bias in the gene. 

### Statistical analysis

Correlation analysis was performed to compare the relationship between nucleotide composition and synonymous codon usage pattern using Spearman’s rank correlation analysis method. A phylogenetic tree was constructed by the neighbor-joining method with a bootstrap of 1000 replicates, based on the Clustal W alignment produced with MEGA software version 5. Cluster analysis was performed using the hierarchical cluster method, and the distances between selected sequences were calculated by the Euclidean distance method. All statistical results were analyzed using Student’s t-test, SPSS software version 11.6 for Windows (*p > 0.05*, no difference; 0.01 < *p* < 0.05, non-significant difference; *p < 0.01*, significant difference).

## Results

### Recombination analysis

**Figure 1:**
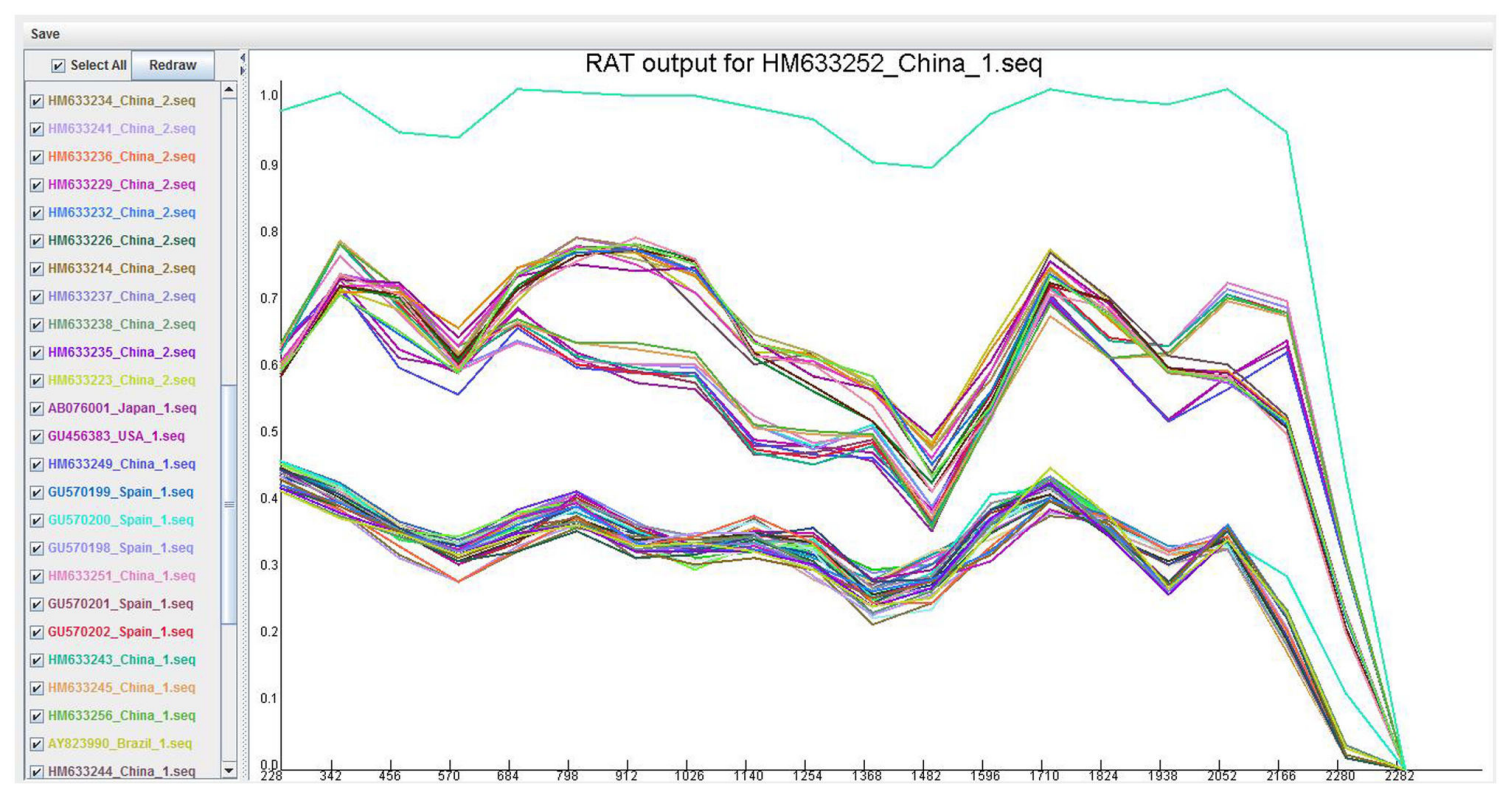
Recombination analysis of TTSuV2 and TTSuV1 sequences using the RAT. The colour of the line on the graph is the same as the colour of its sequence name on the left.

Recombination is believed to influence the efficacy of natural selection on codon usage [30]. A single recombinant sequence present in an alignment can seriously influence the branch order and branch length of the trees generated using standard phylogenetic methods [36]. Therefore, it was necessary to exclude any TTSuV2 and TTSuV1 sequences found to be recombinant from further analysis. Recombination analysis of a nucleotide sequence alignment including all 41 TTSuV2 sequences and 29 TTSuV1 sequences was performed using RAT software (Figure 1). The resulting graph provided no evidence for recombination within or between TTSuV2 and TTSuV1 sequences. However, the graph indicated that the sequences diverged at nucleotide position 2282 into branches corresponding to TTSuV2 and TTSuV1.

The 41 TTSuV2 sequences were further analyzed for codon usage bias and the synonymous codon usage pattern between TTSuV2 and TTSuV1 (previously analyzed) [29] was compared, as described in the following sections. 

### Compositional properties

The nucleotide content of the TTSuV2 genomes is provided in Table 2. In the CDSs from the 41 genomes, A and G occurred more frequently than C and T. A occurred most frequently at the third codon position (average A_3S_% = 41.77%) and T occurred the least frequently (average T_3S_% = 27.67%). The overall nucleotide composition and the composition at the third codon position in TTSuV2 genomes suggest that compositional constraint might be influencing the codon usage pattern of this genome. The GC% of TTSuV2 genomes (42.9% to 46.7%, average 45.1%) is lower than for other vertebrate DNA viruses. The GC_3S_% ranged from 43.2% to 48.2% with a mean value of 46.2%. Due to this compositional constraint, it was expected that A would occur most frequently at the third codon position in TTSuV2 genomes. 

**Table 2:** Nucleotide content of 41 TTSuV2 genomes (%).

**No.**	**T(U)**	**T(U)_3S_**	**C**	**C_3S_**	**A**	**A_3S_**	**G**	**G_3S_**	**GC**	**GC_3S_**	**ENC**
1	20.54	27.81	21.41	33.11	36.33	41.96	21.72	28.10	45.0	46.0	54.55
2	20.22	26.48	22.54	32.61	35.60	43.24	21.64	26.19	45.9	45.3	55.20
3	20.40	27.06	22.41	33.55	34.88	39.46	22.31	29.01	46.7	48.0	57.31
4	20.40	27.06	22.41	33.55	34.88	39.46	22.31	29.01	46.7	48.0	57.31
5	20.75	27.89	21.67	33.33	35.44	40.17	22.14	27.76	45.6	46.5	57.86
6	20.44	27.91	21.83	33.41	35.90	40.40	21.83	28.33	45.5	46.6	58.18
7	20.50	28.07	21.57	32.89	35.74	40.52	22.19	28.17	45.6	46.2	58.16
8	20.39	27.91	21.88	33.41	35.90	40.40	21.83	28.33	45.6	46.6	58.18
9	20.34	27.31	21.37	33.26	36.47	41.72	21.83	28.47	45.1	46.5	53.76
10	20.03	27.63	21.67	32.89	36.47	41.69	21.83	27.25	45.6	45.8	56.04
11	20.03	27.03	21.52	33.19	36.11	41.33	22.34	27.88	45.8	46.4	55.66
12	20.96	28.54	21.47	32.68	35.44	39.91	22.14	28.13	45.4	46.2	58.18
13	21.50	27.29	21.34	31.99	37.02	44.04	20.15	28.54	43.4	45.1	54.69
14	19.93	28.05	21.52	33.71	36.67	41.83	21.88	27.99	45.4	46.1	55.37
15	20.44	28.28	21.06	34.39	37.03	41.31	21.47	27.62	44.6	46.5	55.04
16	19.81	28.28	21.83	33.71	36.58	40.40	21.78	27.92	45.8	46.5	57.14
17	20.09	26.23	21.79	34.75	36.17	41.24	21.95	29.17	46.1	48.1	57.56
18	19.88	28.41	21.73	33.33	36.72	41.74	21.67	26.97	45.5	45.5	56.51
19	20.24	28.07	21.62	32.68	35.95	40.35	22.19	29.02	45.8	46.6	57.03
20	19.93	28.05	21.62	33.71	36.52	41.65	21.93	27.86	45.5	46.1	55.55
21	20.29	27.95	21.57	32.53	35.90	40.35	22.24	28.78	45.9	46.5	56.86
22	20.40	27.33	20.87	31.89	37.90	43.16	20.82	30.00	44.2	45.9	57.78
23	20.66	26.29	21.23	33.71	36.12	40.80	22.00	30.26	45.2	48.0	56.84
24	20.34	28.48	21.06	33.86	36.88	41.31	21.73	28.06	44.8	46.4	54.86
25	21.81	28.51	20.92	31.63	36.86	45.07	20.40	26.82	42.9	43.8	53.98
26	19.99	26.29	22.00	35.06	36.01	40.92	22.00	29.10	46.3	48.2	57.24
27	20.59	28.16	21.57	33.92	36.07	40.77	21.78	28.06	45.3	46.7	54.41
28	21.09	27.25	21.34	31.21	37.42	44.59	20.16	28.12	43.4	44.4	55.97
29	20.56	26.15	21.84	33.63	35.55	41.16	22.05	28.95	46.1	47.5	56.81
30	20.34	28.28	21.11	34.39	36.93	40.99	21.62	27.79	44.9	46.7	54.93
31	21.45	26.81	21.14	31.21	36.90	43.72	20.52	28.50	43.7	44.9	56.01
32	20.08	26.67	21.57	33.11	36.31	42.54	22.03	28.07	45.3	46.0	56.14
33	20.38	29.12	21.57	32.28	37.31	43.51	20.74	26.85	44.7	44.1	56.39
34	20.77	27.27	21.13	31.82	37.64	42.89	20.46	30.56	44.1	46.2	56.61
35	21.19	29.36	21.19	30.91	37.16	43.94	20.47	26.42	43.7	43.2	56.11
36	21.18	28.47	20.40	31.48	37.85	42.66	20.56	31.39	43.5	45.9	57.41
37	21.08	28.37	20.46	31.63	38.21	43.22	20.25	31.22	43.2	45.8	56.27
38	20.43	27.33	21.47	33.78	36.38	40.67	21.72	29.15	45.2	47.3	54.02
39	19.88	26.93	21.93	33.55	36.67	42.83	21.52	27.23	45.4	45.8	54.49
40	20.40	28.92	21.64	32.96	37.35	43.12	20.61	26.59	44.4	44.4	56.01
41	20.14	27.11	21.79	33.78	36.27	41.63	21.79	28.85	45.7	47.1	56.25
mean	20.48	27.67	21.52	33.04	36.46	41.77	21.53	28.35	45.09	46.18	56.21

The ENC values of these TTSuV2 genomes were much higher than genomes of other DNA viruses, varying from 55.20 to 58.18 with a mean value of 56.21. This result indicates that codon usage bias is not remarkable in TTSuV2 genomes and is apparently maintained at a stable level.

### Codon usage in TTSuV2

The overall RSCU values for the 59 codons in all 41 TTSuV2 genomes indicated that A and C occurred most frequently at the third codon position (i.e., GUA for Val, GCA for Ala, CAA for Gln and AAC for Asn) as shown in Table 3. In addition, the CCU, ACU and UAU codons, encoding Pro, Thr and Tyr, respectively, occurred more frequently than the other synonymous codons for these amino acids. Two codons encoding Arg, CGA and CGC, also occurred more frequently than their synonymous codons. These results support the hypothesis that compositional constraint is a major contributing factor in codon usage pattern in TTSuV2 genomes.

**Table 3:** RSCU values of codons in TTSuV2, TTSuV1 and swine.^a^

**AA** ^b^	**Codon**	**RSCU**	**TTSuV1**	**SUS** ^c^	**AA** ^b^	**Codon**	**RSCU**	**TTSuV1**	**SUS** ^c^
Phe	UUU	1.00	0.76	**1.11**	Tyr	**UAU**	**1.10**	**1.10**	**1.12**
	UUC	1.00	**1.24**	0.89		UAC	0.90	0.90	0.82
Leu	**UUA**	**1.56**	**1.38**	0.65	Ala	GCU	0.81	**1.20**	**1.36**
	UUG	0.85	1.21	0.85		GCC	0.94	1.15	1.22
	CUU	0.90	0.49	1.20		**GCA**	**1.51**	1.15	1.05
	CUC	1.03	0.71	1.12		GCG	0.74	0.50	0.37
	CUA	1.23	0.99	0.56	His	CAU	0.91	0.60	0.97
	CUG	0.44	1.22	**1.62**		**CAC**	**1.09**	**1.40**	**1.03**
Ile	AUU	0.53	0.57	1.06	Gln	**CAA**	**1.01**	0.90	0.85
	AUC	0.95	1.02	**1.11**		CAG	0.99	**1.10**	**1.15**
	**AUA**	**1.51**	**1.41**	0.83	Asn	AAU	0.97	**1.01**	**1.02**
Val	GUU	0.71	0.62	1.11		**AAC**	**1.03**	0.99	0.98
	GUC	0.56	0.40	0.96	Lys	**AAA**	**1.26**	**1.22**	**1.21**
	**GUA**	**1.74**	1.28	0.64		AAG	0.74	0.78	0.79
	GUG	0.99	**1.71**	**1.29**	Asp	GAU	0.67	0.74	0.95
Ser	UCU	0.82	0.75	**1.34**		**GAC**	**1.33**	**1.26**	**1.05**
	UCC	1.35	0.72	1.20	Glu	**GAA**	**1.26**	**1.08**	**1.09**
	**UCA**	**1.65**	**1.49**	1.00		GAG	0.74	0.92	0.91
	UCG	0.53	0.56	0.31	Cys	UGU	0.67	0.55	**1.06**
	AGU	0.55	1.39	0.93		**UGC**	**1.33**	**1.45**	0.94
	AGC	1.10	1.10	1.22	Arg	CGU	0.58	0.51	0.55
Pro	**CCU**	**1.58**	**1.21**	**1.26**		CGC	0.39	0.93	0.65
	CCC	0.25	0.72	1.08		CGA	0.50	0.63	0.54
	CCA	1.48	1.11	1.23		CGG	0.64	0.55	0.74
	CCG	0.69	0.96	0.43		**AGA**	**2.16**	1.34	**1.86**
Thr	**ACU**	**1.31**	1.01	1.19		AGG	1.73	**2.05**	1.67
	ACC	1.01	**1.23**	1.23	Gly	GGU	0.49	0.74	0.81
	ACA	1.06	1.21	**1.24**		GGC	0.66	0.99	1.08
	ACG	0.63	0.55	0.34		**GGA**	**2.07**	**1.31**	**1.18**
						GGG	0.78	0.95	0.94

^a^ The preferred codons for each amino acid is displayed in bold.

^b^ AA is the abbreviation of Amino Acid.

^c^ SUS is swine.

**Figure 2:**
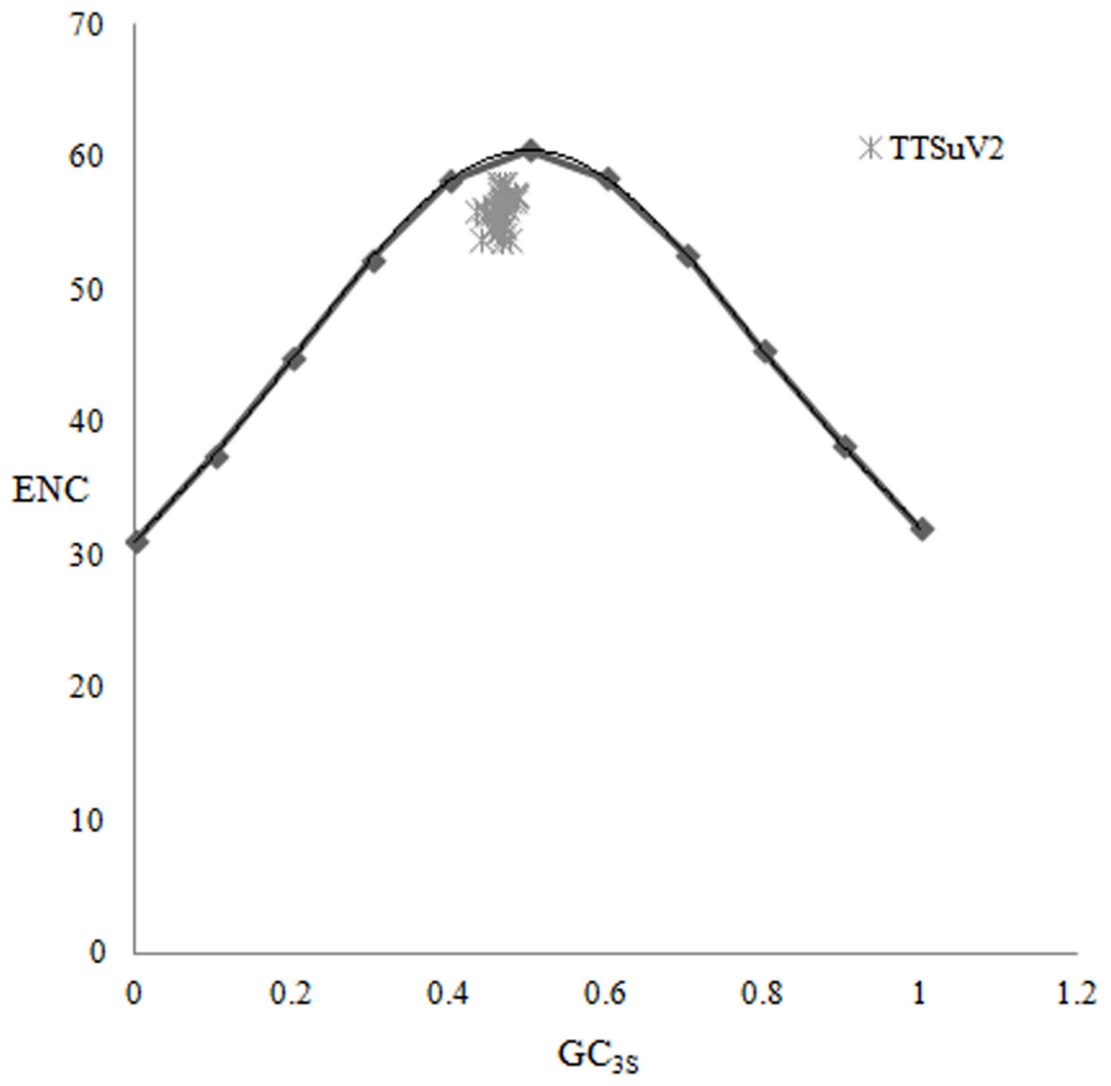
Distribution of the ENC values and GC content at synonymous codon third position (GC_3S_). The curve indicates the expected codon usage if compositional constraint alone account for codon usage bias.

For TTSuV2 sequences, ENC was plotted against both the GC content at the third synonymous codon position (GC_3S_%) and the expected ENC values, as determined by CodonW analysis (Figure 2). All actual codon usage indices were lower than expected, although differences were small. In addition, a positive correlation (r = 0.316, 0.01 < *p* < 0.05) between GC_3S_ and ENC values was found. These results taken together support the conclusion that factors other than compositional constraint under mutation pressure (the major factor accounting for codon usage bias) have influenced TTSuV2 evolution. 

### COA of codon usage

To investigate RSCU variation, COA was performed using the 41 TTSuV2 genomes as a single dataset. As described in the "Materials and methods" section, the distribution of genes on the COA axis was used to identify the source of the variation among a set of multivariate data points. A major trend in the first axis (*f*_1_’) accounted for 16.91% of total synonymous codon usage variation, and the second major trend in the second axis (*f*_2_’) accounted for 13.72% of the total variation (data not shown). 

**Figure 3:**
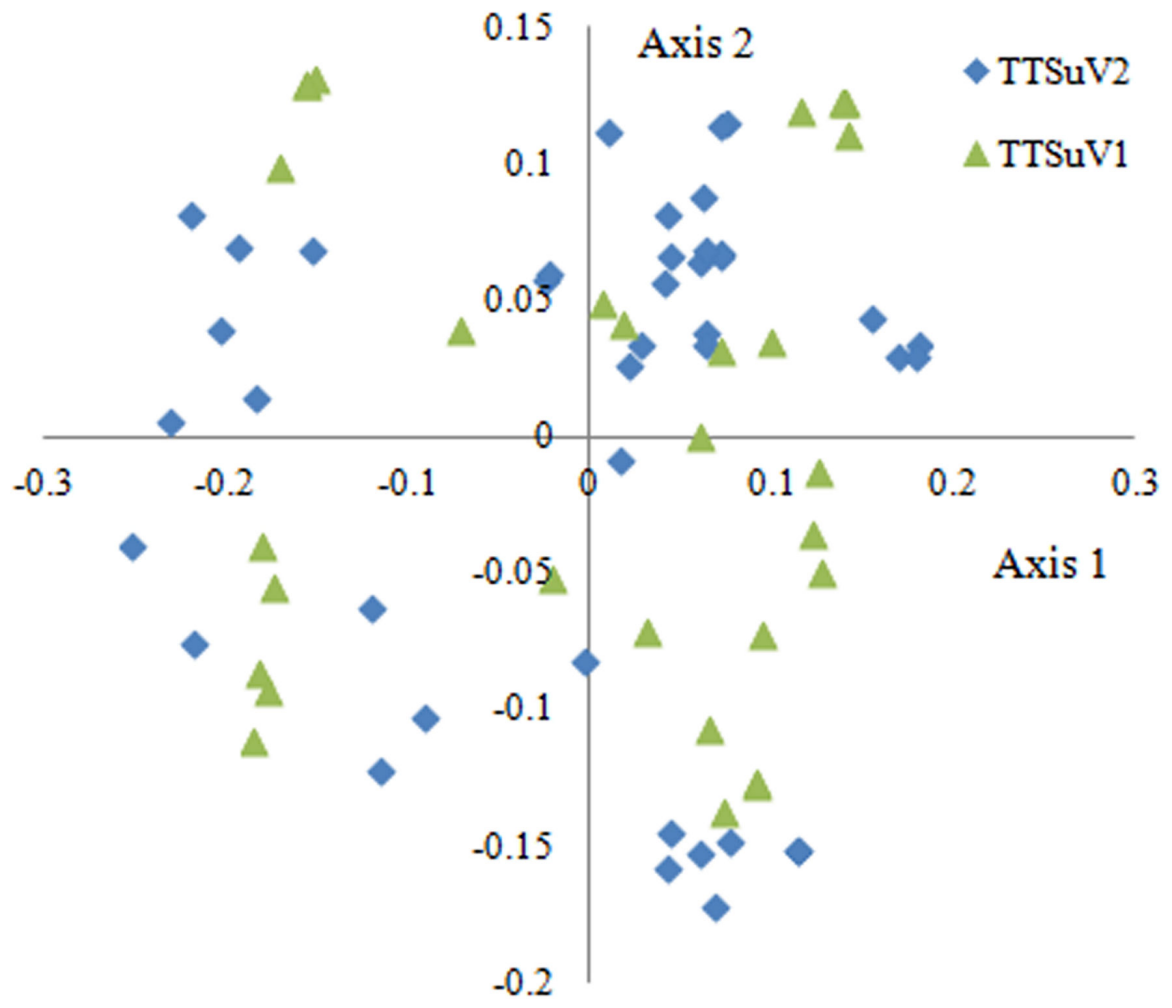
Correspondence analysis of codon usage patterns of TTSuV2 and TTSuV1.

COA was performed for TTSuV1 and TTSuV2 genomes separately and the first two axes of the plots are shown in Figure 3. Although TTSuV1 and TTSuV2 genes occupied all four quadrants of the rectangular coordinate system, the points were generally separated from each other. This result reveals that variation in codon usage might be one of the factors driving the observed aspect of TTSuV evolution.

### Effect of mutational bias on codon usage variation

To explore whether the evolution of codon usage bias in TTSuV2 CDS had been driven by mutation pressure alone or whether translation selection from its host has also contributed, we first compared the correlation between general nucleotide composition (A%, T%, G%, C%, GC%) and nucleotide composition at the third codon position (A_3S_%, T_3S_%, G_3S_%, C_3S_%, GC_3S_%) using the Spearman’s rank correlation analysis method (Table 4). A significant positive correlation was observed between A% and A_3S_% (r = 0.761, *p < 0.01*), C% and C_3S_% (r = 0.392, 0.01 < *p* < 0.05), GC% and GC_3S_% (r = 0.645, *p < 0.01*) and significant negative correlation was observed for most of heterogeneous nucleotide comparisons. Taken alone, these results suggest that compositional constraints under mutation pressure determine the codon usage pattern for TTSuV2. However, a significant positive correlation between G% and C_3S_% (r = 0.434, *p < 0.01*), GC% and T_3S_% (r = 0.434, *p < 0.01*) and no correlation between T% and T_3S_% (r = 0.175, *p > 0.05*), G% and G_3S_% (r = 0.171, *p > 0.05*) suggest that natural selection from its host might have played an appreciable role in determining the codon usage pattern of this virus.

**Table 4:** The correlation analysis between A, T, G, C, GC contents and A_3S_, T_3S_, G_3S_, C_3S_, GC_3S_ contents in TTSuV2 CDS.^a^

	A_3S_%	T(U)_3S_ %	G_3S_ %	C_3S_ %	GC_3S_ %
A%	0.761^**^	0.378^*^	-0.086^NS^	-0.364^*^	-0.616^**^
U%	0.238^NS^	0.175^NS^	0.234^NS^	-0.52^**^	-0.237^NS^
G%	-0.805^**^	-0.391^*^	0.171^NS^	0.434^**^	0.664^**^
C%	-0.458^**^	-0.393^*^	-0.139^NS^	0.392^*^	0.378^*^
GC%	-0.710^**^	0.434^**^	0.078^NS^	0.505^**^	0.645^**^

a Value in this table is the P-value of correlation analysis.

NS, non-significant (*p*>0.05).

* *0*.*01<p<0*.*05*.

** *p*<0.01*.*

Furthermore, G + C content at the first and second codon positions (GC_1_% and GC_2_%) was compared with the G + C content at the third codon position (GC_3_%). A highly significant correlation was observed between GC_1_% with GC_2_% (r = 0.551, *p < 0.01*), GC_3_% (r = 0.699, *p < 0.01*), and GC_2_% with GC_3_% (r = 0.490, *p < 0.01*). Since the effects were present at all codon positions, the results further support the hypothesis that nucleotide constraint under mutation pressure was a main determinant for synonymous codon usage pattern in TTSuV2.

COA was also performed for the first two principle axes (*f*_1_’ and *f*_2_’) and A%, T%, G%, C%, GC%, A_3S_%, T_3S_%, G_3S_%, C_3S_%, GC_3S_% (Table 5). The first principle axis (*f*_1_’) exhibited a significant positive correlation with G%, C%, GC%, C_3S_%, GC_3S_% and a negative correlation with A%, A_3S_%. It was interesting to note that, except G_3S_% (r = –0.357, 0.01 < *p* <0.05), the second principle axis (*f*_2_’) had no correlation with any nucleotide content. These results further support the conclusion that composition constraints under mutational bias is an important factor determining synonymous codon usage pattern in TTSuV2, and but that other factors, such as natural selection, contributed. 

**Table 5:** The correlation analysis between the first two axes and nucleotide contents in TTSuV2 CDS.^a^

	A%	T(U)%	G%	C%	GC%	A_3S_%	T(U)_3S_%	G_3S_%	C_3S_%	GC_3S_%
*f*_1_’	-0.631^**^	-0.367^*^	0.614^**^	0.493^**^	0.552^**^	-0.619^**^	-0.260^NS^	0.054^NS^	0.664^**^	0.608^**^
*f*_2_’	0.071^NS^	-0.014^NS^	-0.023^NS^	-0.233^NS^	-0.236^NS^	0.017^NS^	0.270^NS^	-0.357^*^	-0.093^NS^	-0.286^NS^

a Value in this table is the P-value of correlation analysis.

NS, non-significant (*p*>0.05).

* *0*.*01<p<0*.*05*.

** *p*<0.01

### Relationship between TTSuV and host codon usage patterns

In the ENC plot (Figure 2), most points were near to and under the expected curve, which suggested that other factors contributed to codon usage bias in addition to mutation pressure. To examine this further, a comparative analysis of RSCU values was performed for TTSuV2, TTSuV1 and swine, the natural host for this virus. We found that the codon usage pattern of TTSuV2 was mostly coincident with that of TTSuV1 and that the similarity between the viruses and the host was low. In particular, except for CCU encoding Pro and UAU encoding Tyr, all the preferentially used codons in TTSuV2 and TTSuV1 had an A or C in the third codon position: UUA for Leu, AUA for Ile, UCA for Ser, CAC for His, GAC for Asp and UGC for Gly (Table 3). In contrast, most frequent codons in swine had a T or A at the third codon position. Although some codons frequent in swine, such as CAC for His, AAA for Lys, GAC for Asp and AAA for Glu, were also frequent in TTSuV2 and TTSuV1, the high frequency codons in swine (CUG for Leu, UCU for Ser, UGU for Cys) were generally low frequency codons in TTSuV2 and TTSuV1. It was worth noting that the similarity to swine was higher for TTSuV1 than it was for TTSuV2. The RSCU values of synonymous codons in TTSuV1 and swine, including GUG for Val, GCU for Ala, CAG for Gln, AAU for Asn, were clearly different than TTSuV2 values. This suggests that TTSuV1 might have adapted to its host under natural selection to some degree for improved translation efficiency and that selection pressure from the host had less effect on codon usage pattern of TTSuV2.

### Phylogenetic and cluster analysis

**Figure 4:**
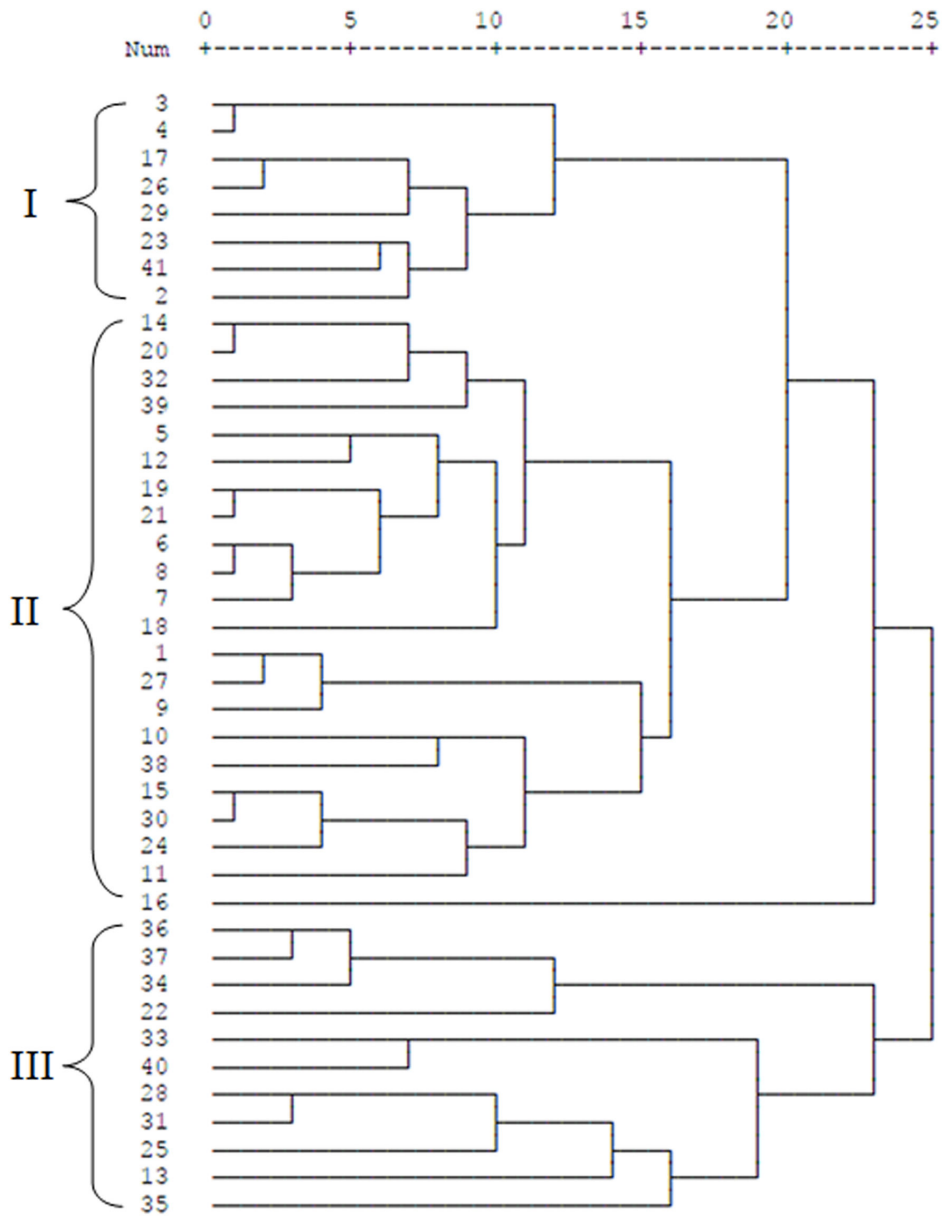
Cluster tree result of 41 TTSuV2 genes based on hierarchical cluster method.

A cluster tree was generated with the RSCU values from all 41 TTSuV2 genomes using a hierarchical cluster method. As shown in Figure 4, the TTSuV2 CDS were divided into three main lineages (I–III). Lineage I comprised two strains isolated from the USA, one from Germany and five from China. Twenty-two strains isolated from Brazil, Spain and China were grouped into Lineage II. Lineage III was comprised of strains isolated from China only. Some genes from different isolates were classified into the same lineage, while others genes from the same isolate were classified into different lineages; thus lineage did not correspond well with geographical distribution.

**Figure 5:**
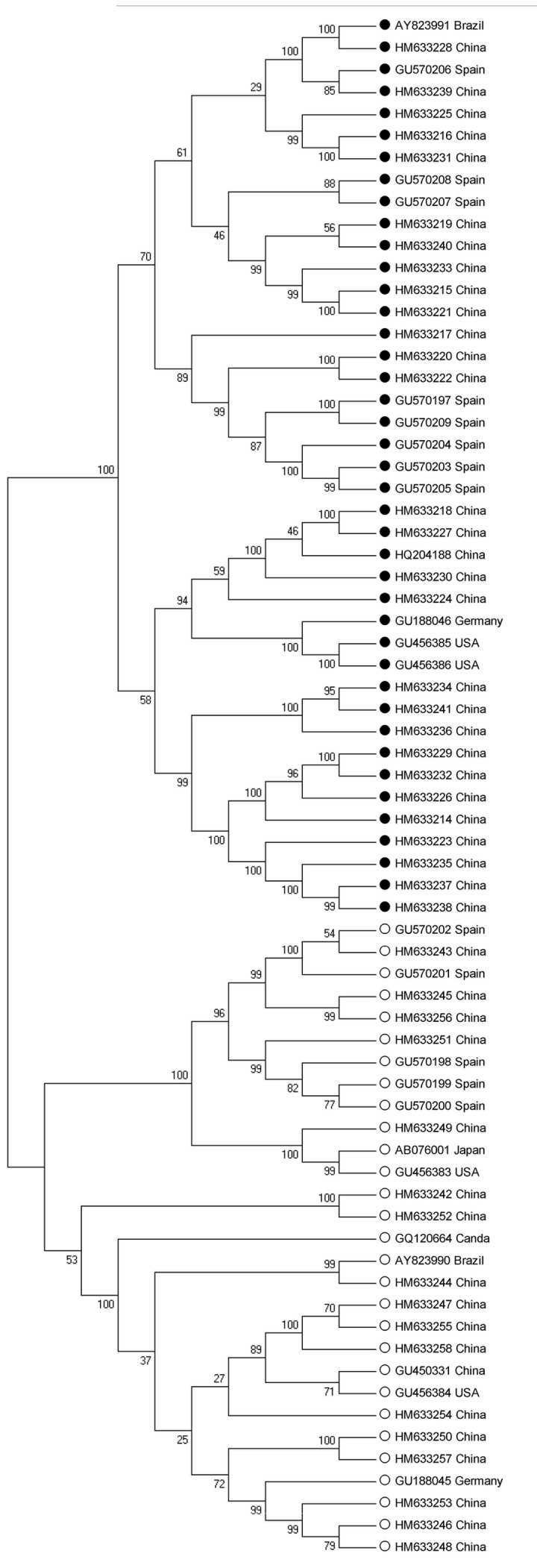
Phylogenetic tree of 41 TTSuV2 sequences and 29 TTSuV1 sequences. ● represents TTSuV2 and ○ represents TTSuV1.

The phylogenetic analysis of all 41 TTSuV2 (black dots) and 29 TTSuV1 sequences (white dots) was performed to determine the conservation and variation of codon usage pattern within TTSuV lineages (Figure 5). The two major branches of the resulting phylogenetic tree corresponded to TTSuV2 and TTSuV1, and each branch had several minor branches. Thus, phylogenetic analysis of the two viruses did not reveal correlations between sequence differences and geographical distribution.

## Discussion

TTSuV is an emerging small DNA virus, widely distributed in pig-farming countries. Although reports implicate TTSuV in co-infection with other diseases, in depth studies on molecular characteristics and pathogenic mechanism are lacking [37,38]. Synonymous codon usage is a well established technique for analyzing genetic information from viral genomes. Most codon usage studies have focused on higher organisms or microorganisms with large genomes and viruses that pose a great threat to human health, such as human immunodeficiency virus, human bocavirus [39], hepatitis virus [40] and Influenza A virus [41]. Results from analyzing codon usage bias in TTSuV genomes are expected to contribute to the knowledge of the characteristics and molecular evolution of this virus. This report furthers our investigation of synonymous codon usage variation in TTSuV1 and provides the first analysis of TTSuV2.

Recombination is an important event in viral evolution and epidemiology [42]. It is interesting to note that recombinant viruses appear to be highly pathogenic, suggesting that recombination events either preserve or increase the pathogenicity of the original strains. Various studies have demonstrated that natural inter- and intra-genotypic recombination occurs frequently in viruses, as shown for highly pathogenic porcine reproductive and respiratory syndrome viruses [43], PCV2 [44], humane enterovirus 71 [45], and rabbit haemorrhagic disease virus [46]. Thus, before analyzing codon usage bias for TTSuV2, we first conducted recombination analysis of 41 TTSuV2 sequences and 29 TTSuV1 sequences, and found no evidence for recombination between the two viruses (Figure 1).

In this study, we analyzed synonymous codon usage bias in TTSuV2 CDS, as well as the relationship between codon usage patterns of TTSuV2 and TTSuV1. Most frequent codons in both TTSuV2 and TTSuV1 had A or C at the third codon position. Mean ENC values for H5N1 influenza A virus [47], severe acute respiratory syndrome [48] and human bocavirus [39], reported as 50.91, 48.99 and 44.45, respectively, are lower than the ENC values for TTSuV2 and TTSuV1 (56.21 and 56.46, respectively), indicating a relatively low codon usage bias for these two viruses. Codon usage patterns for TTSuV2 and TTSuV1 were remarkably similar. In addition, no significant relationship was found between the codon usage pattern of TTSuV2 and its host; although TTSuV1 codon usage was comparatively more similar to swine than that of TTSuV2 (Table 3). This observation might be the result of genome composition evolution and dynamic processes of mutation and selection that enabled the TTSuV1 virus to escape the antiviral cell responses and adapt its codon usage to its host environment [49]. 

In this study, nucleotide frequency at the third codon position of synonymous codons correlated to general composition for some codons but not for others (Table 4). The GC content was similar at all codon positions in TTSuV2 genomes, presumably as a result of mutational pressure. In addition, the general correlation between codon usage bias and composition constraint suggest that mutational pressure was an important factor determining codon usage in TTSuV2, as seen in the highly significant correlation between GC_1_%, GC_2_% and GC_3_% (*p < 0.01*), and remarkable correlation between *f*_1_’ values with respect to A%, G%, C%, GC%, A_3S_%, G_3S_%, GC_3S_% (*p<0.01*) (Table 5). Furthermore, in all ENC plots, values for TTSuV2 genomes were below the expected curve (Figure 1). Taken together, the above evidence indicates that compositional constraint under mutational pressure significantly contributed to the variation of synonymous codon usage in TTSuV2 genomes. 

Natural selection has been shown to influence the synonymous codon usage pattern in viruses [50] and this conclusions is supported by this study. First, although the GC_3S_% for the TTSuV2 genome is lower than average (46.20%), the most frequent codons had A or C at the third codon position (Table 3). Second, a significant positive correlation existed between G% and C_3S_%, and GC% and T_3S_% (*p < 0.01*), whereas no correlation was detected between T% and T_3S_% or G% and G_3S_% (*p > 0.05*) (Table 4). Except G_3S_%, no correlation was found between *f*_2_’ values and A%, T%, G%, C%, GC%, A_3S_%, T_3S_%, C_3S_% or GC_3S_% (*p > 0.05*) in this study (Table 5). Third, most points in the ENC plot were close to the expected curve, although all were below it (Figure 2). The above evidences suggests that, in addition to mutation pressure, natural selection played an important role in determining codon usage bias for TTSuV2 genomes as well. Thus, codon bias in the TTSuV2 genome is multi-factorial. We believe that these characteristics of TTSuV2 genomes might have conferred adaptive advantage resulting in a highly efficient dissemination of this virus through different modes of transmission. 

The analysis of TTSuV genome sequences identified two genetically distinct species, TTSuV1 and TTSuV2. COA was performed to detect possible codon usage variation between these two viruses. Unexpectedly, the distribution of the two viruses showed that genetically distinct species were distantly located in the plane defined by the first two axes of the analysis (Figure 3). A cluster tree analysis based on the RSCU values of TTSuV2 genomes revealed that geographic factors failed to correspond to the codon usage pattern of this virus (Figure 4). Further, the phylogenetic tree had two major branches corresponding to the two different species, and no specific geographical correlation was detected in this analysis (Figure 5). It seems likely that, given extensive international communication and various modes of transmission for this virus, geographical distance is a weak factor in the distribution of TTSuV2 in different countries. 

In summary, our investigation of synonymous codon usage pattern in TTSuV2 CDS revealed that codon usage bias is not remarkable, possibly representing the interactions between compositional constraint under mutation pressure and natural selection. However, both TTSuV1 and TTSuV2 genomes exhibited significant synonymous codon usage bias favoring A or C at the third codon position, presumably determined by compositional constraint under mutation pressure. Although the analysis of synonymous codon usage does not perfectly reflect the genetic variation of TTSuV2 nor does it distinguish between TTSuV1 and TTSuV2, our results provide an insight into the codon usage variation in TTSuV2 genes that may also facilitate understanding of TTSuV evolution. 
